# Mutual positive engagement with mother is uniquely associated with increases in human infant heart rate variability

**DOI:** 10.1098/rstb.2026.0004

**Published:** 2026-09-24

**Authors:** Drew H. Abney, Stephen J. Sheinkopf, Samantha G. Mitsven, Ronald Seifer, Barry M. Lester, Ed Z. Tronick, Daniel S. Messinger

**Affiliations:** ^1^ Department of Psychology, University of Georgia, Athens, GA 30602, USA; ^2^ Thompson Center for Autism & Neurodevelopment, University of Missouri, Columbia, MO 65201, USA; ^3^ Department of Psychology, University of Miami, Coral Gables, FL 33146, USA; ^4^ Frank Porter Graham Child Development Institute, The University of North Carolina, Chapel Hill, NC 27514, USA; ^5^ Department of Pediatrics, The Warren Alpert School of Medicine of Brown University, Providence, RI 02903, USA; ^6^ University of Massachusetts, Boston, MA 02125, USA

**Keywords:** emotion regulation, heart rate variability, infant–caregiver interaction

## Abstract

The ability of human infants to dynamically adapt their physiological states to changing social demands is critical for the development of emotion regulation. We investigated infant parasympathetic dynamics related to emotion regulation—changes in respiratory sinus arrhythmia (RSA)—using a large sample of 974 at-risk infant–mother dyads (infant age: 4 months). Infant RSA was elevated during bouts of positive engagement matching with their mothers, characterized by mutual positive engagement. Specifically, infant RSA began to increase more than 5 seconds before the onset of bouts of positive engagement matching and remained elevated for at least 4 seconds after bout onsets. These periods were substantially extended when the bouts began with positive expressions from the mother. Strikingly, there were no increases in infant RSA before or during bouts of either infant or mother solo positive engagement. Findings from these exploratory analyses suggest the possibility of bi-directional associations between ongoing social interactions and infant parasympathetic reactivity in which positive mother expressions support increases in infant RSA that in turn support prolonged infant engagement in mutual positivity. Thus, early social interaction is not simply an elicitor of infant positive expressions. Instead, mutual positive expressivity may facilitate infant neurophysiological changes that underlie emotion regulation.

This article is part of the theme issue ‘Mechanisms, development, phylogeny and functions of emotional expressions’.

## 1. Introduction

Infants and parents engage in frequent bouts of interaction that include the exchange of facial and vocal emotional expressions. Decades of research indicate that large-scale changes in context such as the presence and absence of opportunities for social interaction affect infant physiological functioning [1–6]. However, little is known about dynamic linkages between infant physiological activity and infant–parent behavioural interaction at shorter time scales. One medium for such linkages is infant interactive emotional expressivity. Infant emotional expressions develop during interaction and are probably linked to physiological changes in arousal and regulation [5,7]. But little is known about the mechanisms and functions of infant emotional expressivity and dyadic emotional interaction in the developing physiological regulatory system. Therefore, in the current study, we investigated how early infant physiological activity changes dynamically during bouts of emotional interaction with a parent.

It is likely that infant–parent interaction bouts are both supported by and impact infant physiological activity. A premise of previous research is that changes in infant physiological activity are linked directly to infant concurrent behaviours, while infant behaviour is, in turn, linked to changes in the social context [8,9]. It is also possible, however, that moment-to-moment changes in the social context—*joint infant–caregiver dyadic behaviour*—are directly associated with changes in infant parasympathetic activity. To distinguish between these possibilities, we asked whether changes in infant physiology are linked to an infant's own emotional expressivity, the mother's emotional expressivity or joint infant–mother expressive activity.

Respiratory sinus arrhythmia (RSA) is a measure of parasympathetic nervous system functioning that indexes variability in heart rate associated with respiration. RSA reflects autonomic flexibility and is a physiological index of infant emotion regulation [9]. A common protocol for studying infant behavioural and physiological changes during the presence, withdrawal and resumption of interaction is the face-to-face/still face (FFSF) paradigm [10]. This paradigm includes an interactive face-to-face ‘play’ episode, a ‘still face’ episode where parents are tasked with not providing contingent responses, and a ‘reunion’ episode that reintroduces contingent face-to-face interaction. A decrease in infant RSA during the stress associated with the termination of interaction (still face episode) is thought to reflect adaptive utilization of attentional and regulatory resources [11]. By contrast, increases in RSA during the ‘play’ and ‘reunion’ episodes are thought to facilitate adaptive interactive functioning [12].

Although there is an expectation that episode-level increases and decreases in RSA during the FFSF paradigm are associated with emotion regulation, we know little about potential associations between increases in RSA and the dynamic social behaviours of infants and their parents. Understanding of the linkages between dynamic changes in infant parasympathetic activity and changes in behaviour is lacking in part because the timescales for measuring physiology and behaviour may be incommensurate. Parasympathetic indices of emotion regulation tend to be aggregations of RSA and behaviour at time epochs of 15 or 30 seconds, which is too coarse to capture dynamic responses occurring at faster timescales [4,13,14]. Consequently, we asked how infant parasympathetic dynamics change during bouts of interactive behaviour with a caregiver at second-to-second timescales. During interaction, an infant's and mother's positive, neutral and negative expressive behaviours create matched (e.g. positive–positive) and mismatched (e.g. positive–neutral) engagement states [15]. We asked how infant RSA was dynamically linked with infant–mother matching states and with unmatched states characterized by, for example, infant solo expressions of positive affect or mother solo expressions of positive affect.

We focused on 4-month-old infants, an age characterized by intense expressions of positive affect during social interaction [16]. Using infant RSA and time-locked micro-coded behavioural data of infant–mother social engagement states, we quantified the proportion of time infant–mother dyads spent in matched and mismatched social engagement states during the three episodes of the FFSF paradigm [10] (analysis 1). We examined differences in infant RSA during bouts of matched and mismatched infant–mother behaviour across these three episodes (analysis 2). Matching and mismatching refer to the joint affective engagement states of infant and mother. Positive engagement matching, for example, occurred if both mother and infant had been coded as simultaneously exhibiting positive expressivity. If one partner exhibited positive engagement and the other did not, this would constitute a mismatch. To examine temporal dynamics, we extracted contiguous empirical (observed) segments of RSA from 10 seconds before to 10 seconds after the onset of bouts of interest (solo infant positive engagement and solo mother positive engagement, infant–mother positive engagement matching and infant–mother neutral engagement matching, and bouts of positive engagement matching led either by infant or by mother positive engagement). For analyses 3–5, we compared each 20-second empirical contiguous RSA series with a random contiguous 20-second RSA series from that same episode and infant (but from outside the empirical bout). Recent previous research has demonstrated dynamic associations between parasympathetic activity and caregiver engagement [17]. Although we had general predictions that RSA would increase around bouts of interactive behaviour, we considered all hypotheses and corresponding analyses to be exploratory. For all analyses that included inferential models, prenatal drug risk and social risk variables were included as covariates. We report and visualize risk factor associations with RSA changes in the supplementary material.

## 2. Results

### (a) Proportion of engagement matching and mismatching across infants and mothers differs as a function of FFSF episodes (analysis 1)

**Figure 1.**
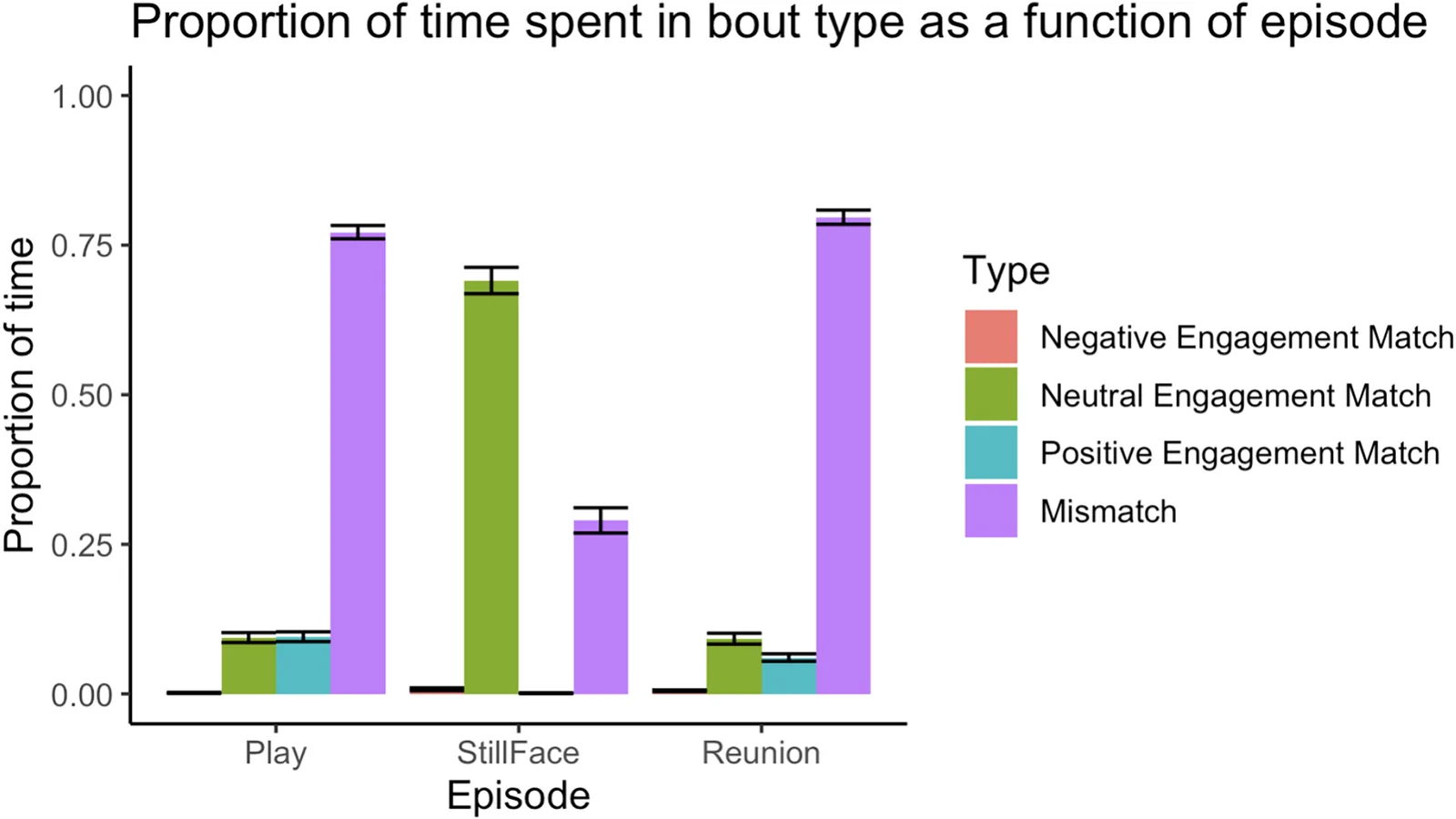
Proportion of time spent in match and mismatch bout types as a function of episode. Confidence intervals represent ±95% intervals.

During the play and reunion episodes, the most common engagement states were mismatches in which infants were in neutral engagement and mothers were in positive engagement, followed by neutral and positive engagement matches [18] (see figure 1). During the still face episode, during which mothers were instructed to remain in a neutral state, infant–mother dyads spent the highest proportion of time in bouts of neutral engagement matching, followed by mismatch bouts in which infants were in negative engagement and mothers were in neutral engagement. It is not surprising to observe very low proportions of positive engagement matching during the still face episode given the nature of the paradigm and instructions to mothers.

### (b) Infant RSA is elevated during bouts of positive engagement matching (analysis 2)

**Figure 2.**
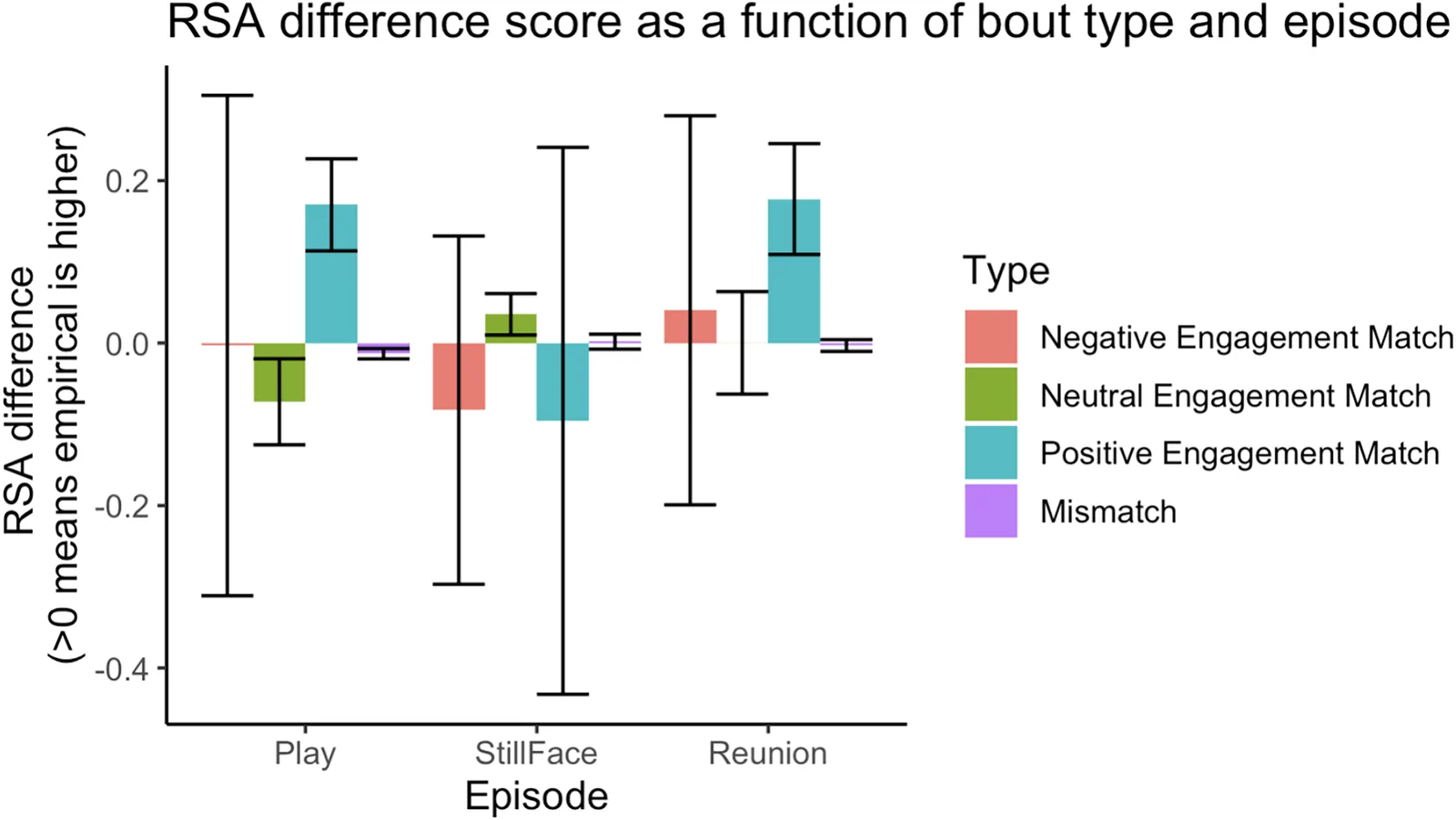
RSA difference scores as a function of match and mismatch bout types and episode. Confidence intervals represent ±95% intervals.

An RSA difference score was computed for each second of each bout (empirical RSA bout minus a randomly chosen RSA bout of similar duration), with a difference score above 0 denoting higher empirical RSA compared with a random bout RSA. Figure 2 shows RSA difference scores as a function of bout type and FFSF episode. Higher difference scores correspond to higher average infant RSA during empirical bouts compared with random bouts. Non-overlapping confidence intervals indicate a statistically significant difference (*p* < 0.05). RSA was higher (i.e. positive difference scores) during bouts of positive engagement matching during the play (*M* = 0.17, ±95% confidence interval (CI) = (0.12, 0.23)) and reunion (*M* = 0.18, ±95% CI = (0.11, 0.25)) episodes. That is, infant RSA was higher throughout bouts of mutual positive engagement. In other words, we observed increased parasympathetic activity that coincided with the onset of bouts of mutual positive engagement.

To further investigate differences in RSA across bout types, we constructed mixed effects models with bout type (positive engagement match, neutral engagement match and negative engagement match) as the main independent variable predicting RSA difference scores. Data collection clinic site, infant race, cumulative prenatal drug risk and cumulative social risk were included as covariates in this and subsequent analyses. We constructed separate models for the play and reunion episodes. Dyad ID was included as a random intercept.

For the play episode, we observed a significant main effect of bout type, *F*(3,4054) = 55.61, *p* < 0.001. Pairwise comparisons using Bonferroni corrections showed that the RSA difference scores were larger and more positive during positive engagement match bouts relative to all other bout types (*p*s < 0.001). RSA difference scores during empirical positive engagement match bouts were greater than difference scores during other bout types. For the reunion episode, we again observed a significant main effect of bout type, *F* (3,3675) = 31.52, *p* < 0.001. Pairwise comparisons using Bonferroni corrections showed that RSA difference scores were higher during positive engagement match bouts relative to neutral engagement match bouts (*p* < 0.001) and mismatch bouts (*p* < 0.001), but not compared with negative engagement match bouts (*p* = 0.09), suggesting higher average infant RSA difference scores compared with all other bout types except for negative engagement matches. The lack of a significant difference in RSA change scores between the positive engagement match bouts and the negative engagement match bouts was probably owing to the high variability in RSA difference scores for negative engagement match bouts, which appeared to be driven by the low frequency of this bout type (see figure 2). Owing to low frequencies of bouts during the still face episode, we did not further investigate parasympathetic dynamics during this episode (see table 1, for bout frequency information).

**Table 1.** Number of bouts for each condition in figures 3–5.

figure	type	episode	# of bouts
figure 3	positive engagement matching	play	2637
		still face	39
		reunion	1658
	neutral engagement matching	play	2363
		still face	2314
		reunion	1952
figure 4	infant positive engagement	play	185
		reunion	147
	mother positive engagement	play	5512
		reunion	4550
figure 5	positive engagement matching (infant led)	play	114
		reunion	360
	positive engagement matching (mother led)	play	2405
		reunion	1452

### (c) Infant RSA in bouts of solo infant positive engagement and solo mother positive engagement (analysis 3)

Logically, of course, positive engagement matches involve the simultaneous positive engagement of both infant and mother. A next step was to determine whether infant RSA changes before and during bouts of positive engagement matching could be explained by the infant's own positive engagement, or by the mother's own positive engagement. Consequently, we examined bouts when infants and mothers were positively engaged, but the other partner was not positively engaged. RSA series were extracted ±10 seconds before and after the onset of bouts of solo infant positive engagement and, separately, solo mother positive engagement for empirical series and randomly selected contiguous 20-second segments. A 20-second segment (±10 seconds before and after the onset of a bout) was chosen because it reflects an approximation of the timescale of RSA response dynamics of human infants: the natural fluctuations of respiration are on the timescale of 5 seconds for human infants, so a 20-second segment reflects a reasonable temporal window to observe changes. No constraints were placed on the randomly selected 20-second segments. Thus, on average, the randomly selected segments are probably to mirror the mixture of matches and mismatches found in a given dyad's behaviour in a particular FFSF episode. This analytic decision was made because of the low frequency of 20-second segments that included no coded bouts. This is a conservative approach to selecting random RSA series segments, as these random segments could contain the contrasting target dyadic behaviour.

RSA series (type: empirical vs. random) were submitted to growth curve models with orthogonal polynomials to the second order (quadratic, linear, intercept). Separate models were fit for RSA series during the play and reunion episodes for the three types of engagement bouts. If second-order polynomials (the quadratic function) best fit the RSA trajectories, this would suggest there was an inflection in relative RSA level at the onset of a particular behavioural bout. For example, a positive quadratic function would suggest that RSA started high, decreased and then increased again. Inversely, a negative quadratic function would suggest that RSA started low, increased and then decreased again.

**Figure 3.**
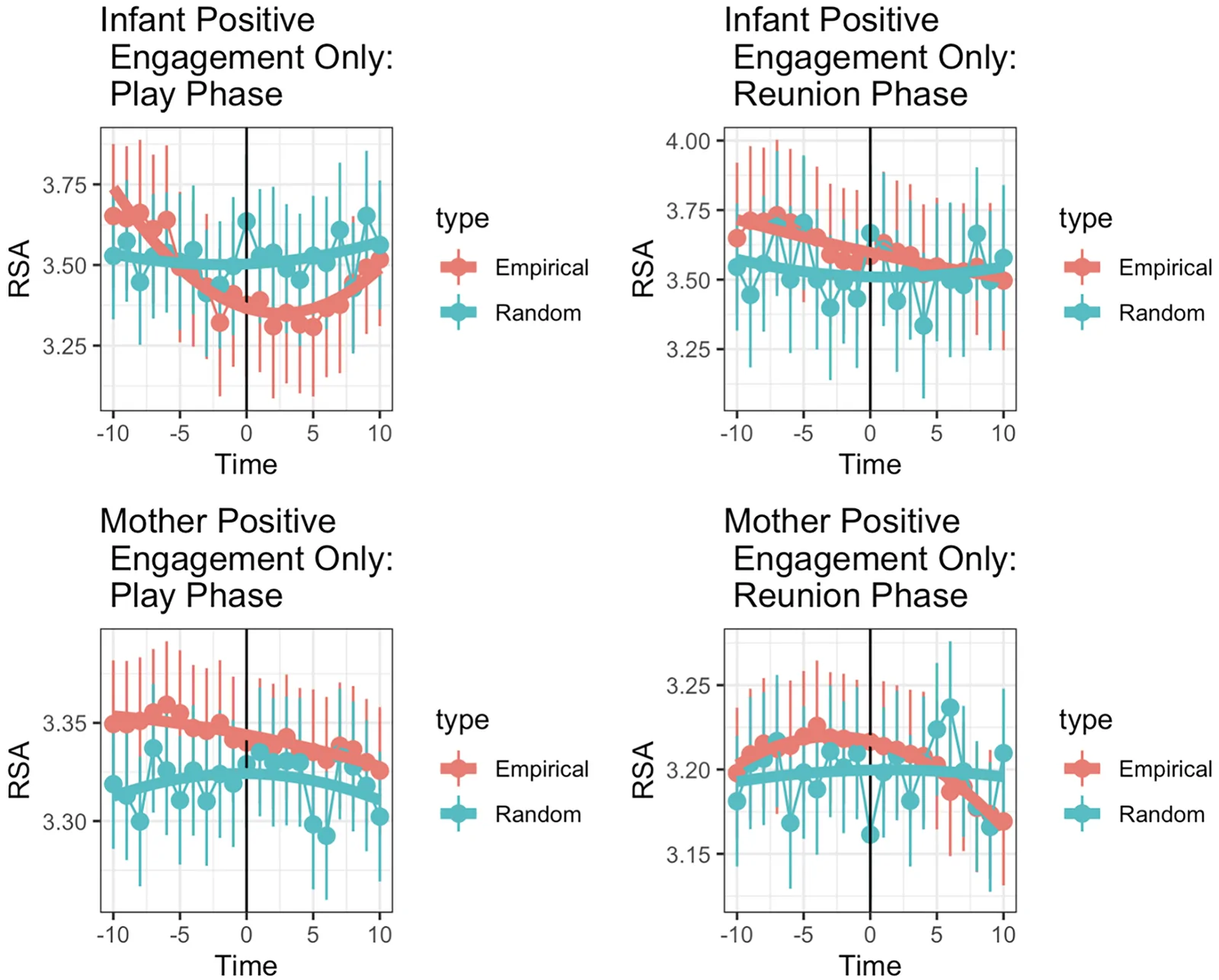
RSA dynamics ±10 s before and after the onset of bouts of infant positive engagement-only bouts (top) and mother positive engagement-only bouts (bottom) for empirical (red) and random (blue) RSA series. Solid vertical lines denote the onset of a bout. Confidence intervals represent ±95% intervals. Thick lines represent fitted lines from growth curve models (without covariates in the model). Note that *y*-axes are not identical across sub-plots.

For infant-only positive engagement bouts (figure 3, top)—when an infant displays positive engagement with no positive engagement matching by the mother—during the play episode—the second-order polynomial contrast was significant, suggesting a stronger positive quadratic function (high RSA to low RSA to high RSA) for the empirical series compared with the random series (*z*-ratio = 2.54, *p* = 0.011). For positive engagement bouts during the reunion episode, the second-order polynomial contrast was not significant, suggesting no difference in the RSA trajectories for empirical or random series, *z*-ratio = −0.41, *p* = 0.685. However, follow-up pairwise comparisons (with Tukey correction) testing differences between average RSA estimates across empirical and random series at each time point suggested no significant differences at any time point for either the play or reunion episodes (see figure 3).

For mother-only positive engagement bouts (figure 3, bottom)—when a mother displays positive engagement, but the infant does not—during the play episode, the second-order polynomial contrast was not significant, *z*-ratio = 1.01, *p* = 0.313. For positive engagement bouts during the reunion episode, the second-order polynomial contrast was significant, suggesting a stronger negative quadratic function (low RSA to high RSA to low RSA) for the empirical series compared with the random series (*z*-ratio = −1.93, *p* = 0.054). However, follow-up pairwise comparisons (with Tukey correction) testing differences between average RSA estimates across empirical and random series at each time point suggested no significant differences at any time point for the play or reunion episodes (see figure 3).

These results suggest that infant RSA does not increase in conjunction with infant positive engagement alone, nor does it increase in conjunction with mother positive engagement alone. Only when infants and mothers are in a matching (i.e. mutual) positive engagement bout do infants show an increase in RSA, suggesting that *reciprocal* bouts of positive engagement are uniquely tied to infant parasympathetic dynamics. Next, we investigate the RSA dynamics around the onset of positive engagement matching.

### (d) Infant RSA changes as a function of the onset of bouts of positive engagement matching (analysis 4)

Having identified elevated RSA during bouts of positive engagement matching (see figure 2), and showing that RSA does not change around bouts of positive engagement without matching (see figure 3), we investigated RSA dynamics before, during and after the onsets of these bout types. We hypothesized larger dynamic changes in RSA and elevated levels of RSA during bouts of positive engagement matching compared with bouts of neutral engagement matching.

RSA series (empirical vs. random) were submitted to growth curve models with orthogonal polynomials to the second order (quadratic, linear, intercept). Separate models were fit for RSA series during the play and reunion episodes for neutral and positive engagement bouts. See table 1 for a breakdown of the number of bouts that were included in each model.

**Figure 4.**
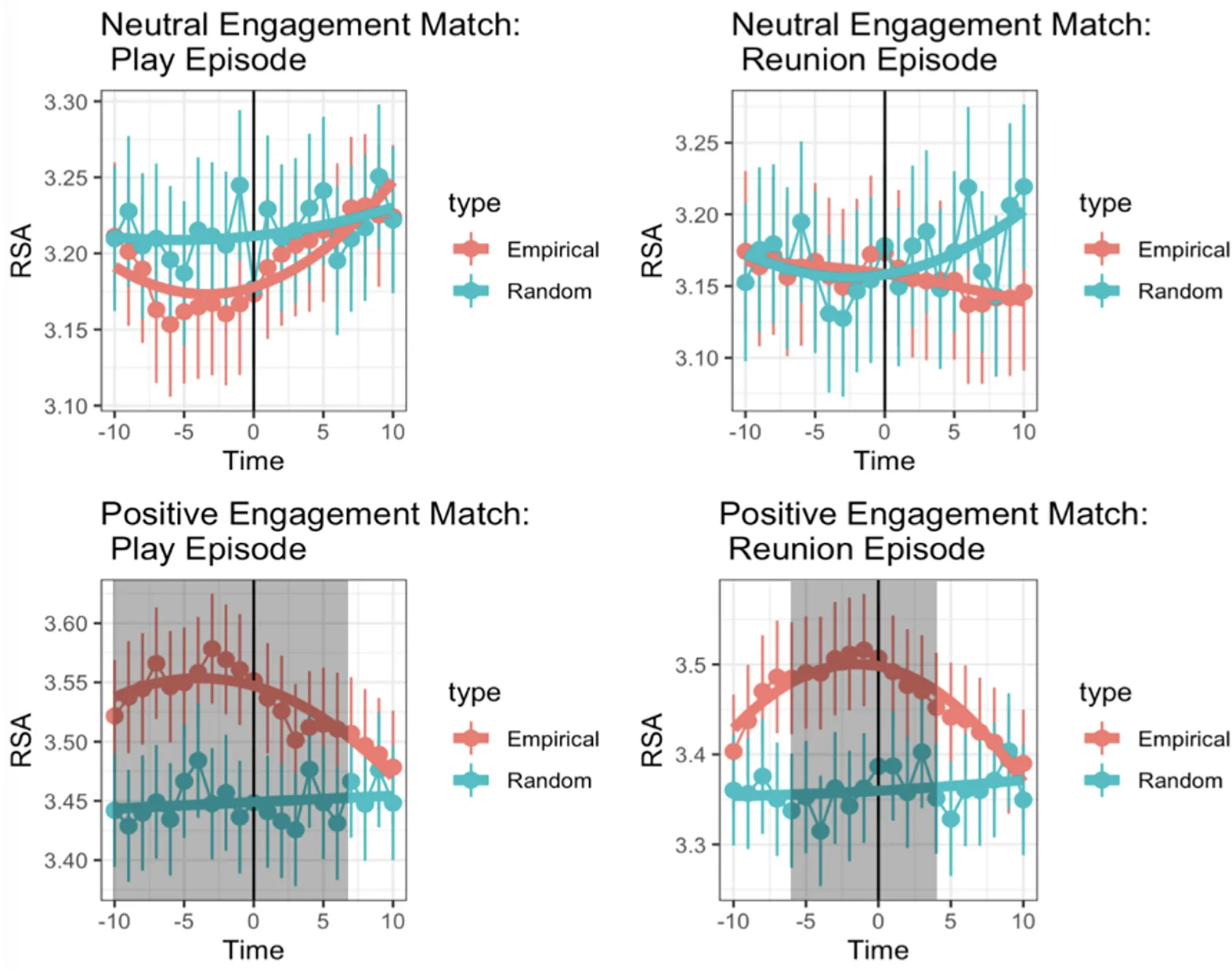
RSA dynamics ±10 s before and after the onset of engagement bouts, as compared with randomly selected time series, for neutral engagement match (top) and positive engagement match (bottom) for empirical (red) and random (blue) RSA series. Solid vertical lines denote the onset of a bout. Confidence intervals represent ±95% intervals. Thick lines represent fitted lines from growth curve models (without covariates in the model). Opaque boxes represent significant (*p* < 0.05) contrasts with Tukey corrections between RSA type at a particular time point as per the growth curve model results (including covariates in the model). Empirical infant RSA is elevated before and after the onset of bouts of positive engagement in the play and reunion phases of the FFSF. Note that *y*-axes are not identical across sub-plots.

For matched neutral engagement bouts (figure 4, top) during the play episode, the second-order polynomial contrast was significant, suggesting a stronger positive quadratic function (high to low to high RSA) for the empirical series compared with the random series (*z*-ratio = 2.50, *p* = 0.013). For matched neutral engagement bouts during the reunion episode, the second-order polynomial contrast was not significant, suggesting no difference in the RSA trajectories for empirical versus random series. Follow-up pairwise comparisons (with Tukey correction) testing differences between average RSA estimates across empirical and random series for neutral engagement bouts at each time point suggested no significant differences at any time point for either the play or reunion episodes (see figure 4).

For matched positive engagement bouts (figure 4, bottom) during the play episode, the second-order polynomial contrast was significant, indicating a stronger negative quadratic function (low to high to low RSA) for the empirical series compared with the random series (*z*-ratio = −2.58, *p* = 0.010). Follow-up pairwise comparisons testing differences between average RSA estimates across empirical and random series at each time point indicated higher RSA for the empirical series from 10 seconds before the onset of a bout until 7 seconds after the onset of a bout (for all differences, *p* < 0.05). For matched positive engagement bouts during the reunion episode, the second-order polynomial contrast was significant, suggesting a stronger negative quadratic function for the empirical series compared with the random series (*z*-ratio = −4.72, *p* < 0.001). Follow-up pairwise comparisons indicated higher RSA for the empirical series from 8 seconds before the onset of a bout until 4 seconds after the onset of a bout. Thus, uniquely for matched positive engagement bouts, infant RSA increases multiple seconds before the onset of the bout and remains elevated after the onset.

### (e) Mothers’ positive engagement that leads to bouts of positive engagement matching is associated with increases in infant RSA (analysis 5)

**Figure 5.**
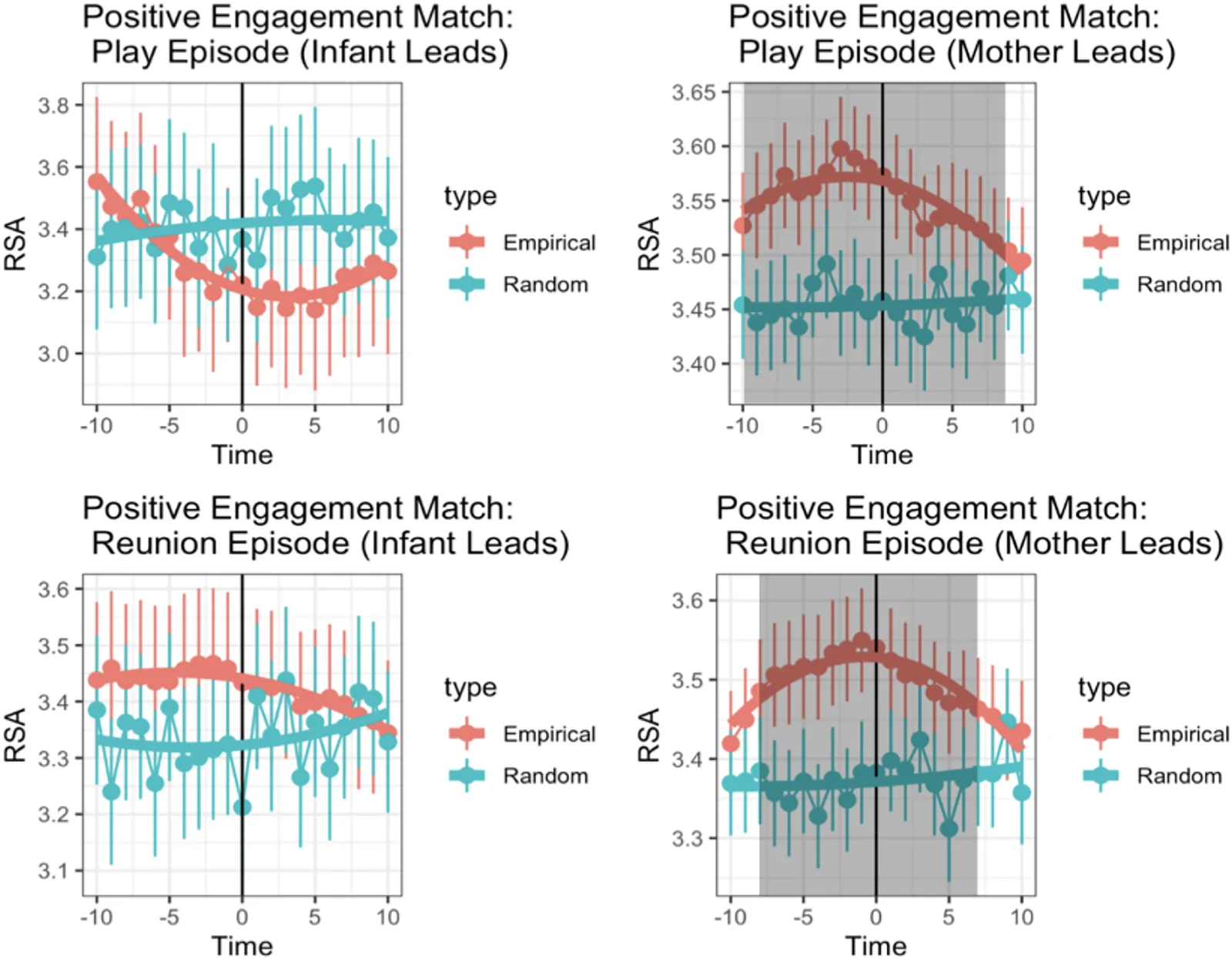
RSA dynamics ±10 s before and after the onset of bouts of positive engagement matching for empirical (red) and random (blue) RSA series across mother-led and infant-led bouts during the play and reunion episodes. Solid vertical lines denote the onset of a bout. Confidence intervals represent ±95% intervals. Thick lines represent fitted lines from growth curve models (without covariates in the model). Opaque boxes represent significant (*p* < 0.05) contrasts with Tukey corrections between RSA type at a particular time point as per the growth curve model results (including covariates in model). Note that *y*-axes are not identical across sub-plots.

To better understand the sources of elevated infant RSA before and during bouts of matched positive engagement, infant RSA dynamics were examined separately depending on whether the infant or the mother led the bout of positive engagement matching. We operationalized the measure of which partner led a bout by defining the timing with which a member of the dyad entered first into the engagement state. For example, if the mother produced positive engagement behaviours and the infant then produced matching positive engagement behaviours, that matched positive engagement bout would be led by the mother. Mothers led bouts of matched positive engagement 95% of the time during the play episode and 75% of the time during the reunion episode. It should be noted that, given the large differences in the frequencies of mother-led and infant-led positive engagement bouts, the power to detect effects was also different, and this should constrain interpretations of results. For mother-led positive engagement-matching bouts during play and reunion episodes, the second-order polynomial contrasts were significant, suggesting a negative quadratic function (low to high to low RSA) for the empirical series compared with the random series (play episode: *z*-ratio = −3.392, *p* < 0.001; reunion episode: *z*-ratio = −4.715, *p* < 0.001). See figure 5 for the pairwise comparisons (with Tukey correction) that showed significant differences between average empirical and random RSA for the majority of seconds before and after the onset of mother-led positive engagement-matching bouts.

For infant-led positive engagement-matching bouts during the play episode, the second-order polynomial contrast was significant, suggesting a positive quadratic function (high to low to high RSA) for the empirical series compared with the random series (*z*-ratio = 2.955, *p* = 0.003). The contrast test for the reunion episode was not significant (*z*-ratio = −1.835, *p* = 0.067). Pairwise comparisons (with Tukey correction) suggested no significant differences between average RSA estimates across empirical and random series at any time point in either the play or reunion episodes.

Results from this set of analyses indicate that elevated infant RSA levels were associated with mother-led positive engagement bouts. This result suggests a probable explanation for the elevated infant RSA levels before the onset of positive engagement-matching bouts. Note that mothers' positive engagement behaviours that were not constrained to involve subsequent positive engagement matching were not associated with increases in infant RSA (figure 3, bottom). By contrast, when mothers produce positive engagement behaviours that are followed by infant positive engagement behaviours, mothers' behaviours are associated with rising infant RSA levels, which probably potentiate the infant's positive engagement behaviours and thus create the emergence of positive engagement-matching bouts (figure 5, right).

#### (i) Cumulative social risk and prenatal drug exposure

Effects in analyses 3–5 controlled for cumulative indices of prenatal drug exposure and social risk. In these analyses, there were no direct effects of either risk index on infant RSA. There was an interaction such that infants with higher levels of prenatal risk showed larger differences between empirical and random RSA time series during matching positive engagement (including mother-led matching positive engagement) during the reunion episode (see supplementary material).

## 3. Discussion

The results from the exploratory analyses in this study challenge our understanding of the interplay between infant physiology, infant behaviour and the social environment. The current results indicate that dyadic interactive states are directly associated with infant parasympathetic activity. Specifically, we observed dynamic increases in infant RSA before, during and after bouts of mother-led positive engagement matching. This finding suggests the influence of dynamic social interactions on infant parasympathetic functioning and broadens our conceptualization of the role of parents in scaffolding infants’ social–emotional experience. That is, matching infant–mother expressions of positive affect is a temporal focal point for changes in an infant's parasympathetic system that may support emotion regulation.

This study reveals a rich natural history in which infant physiology dynamically changes before and during bouts of social engagement with mother during naturalistic social interactions punctuated by a period of maternal non-responsivity (the still face). Strikingly, infant positive engagement alone was not associated with changes in infant physiology, which would have led to the conclusion that elevated RSA was associated with infant positive affect, regardless of social context. Likewise, maternal positive engagement alone was not associated with elevated RSA, which would have suggested infant physiological sensitivity to the partner in the absence of matching behaviour. Instead, mother positive engagement that led to mutual positive engagement (infant matching) was accompanied by a rise in infant RSA. Moreover, increases in infant RSA were evident 7–10 seconds before *and* after mutual positive engagement onset. Our results suggest that infants are able to match maternal positive engagement when this elicits increases in infant parasympathetic activity. Likewise, elevated infant RSA appears to undergird a capacity for flexible social engagement that supports infants’ ability to participate in arousing bouts of mutual positivity. Mutual positive engagement—including infants responding positively to maternal positive engagement—is central to the social development of infant joy [16] and social development, more generally [19–21]. Thus, the second-to-second dynamics of infant parasympathetic activity and dyadic behaviour in a challenging social context—the FFSF—may support functional emotion regulation development.

Occasions when infant RSA is elevated before and after the onset of bouts of positive social behaviours are differentially preceded by their mother's production of positive engagement behaviours. Maternal-led positive engagement matching may facilitate infants entering physiological states that support attention and learning. For example, heart rate changes are related to and dynamically change during bouts of visual attention [22,23], and attention-dependent changes in heart rate and heart rate variability are associated with social communication skills in infants [24]. Indeed, visual attention to faces during infant-directed speech, and lower heart rate during infant-directed speech, are related to later language development [25]. One provocative idea is that the dynamic changes in heart rate variability associated with positive engagement matching may help infants to enter attentional states that support learning [26]. In other words, a potential function of positive engagement matching during development is in supporting cascading effects of the physiological system on attention and learning. Developmental research with older infants and toddlers will be needed to better understand the role of moments of mutual positive expression in infant emotion regulation, social functioning and learning.

Study findings highlight the relationship between physiological responsivity and behaviour at 4 months, an age characterized by the emergence of early social interaction capabilities in human infants [27,28]. At developmental timescales of months and years, individual differences in early-life stress can exert effects on the autonomic nervous system, specifically the parasympathetic nervous system, indexed by RSA [29–32]. High-resting RSA is generally thought to reflect the capacity to be responsive to environmental stressors and may reflect protective mechanisms [1,33–36]. We expand this conceptualization by finding that during face-to-face interaction, a particular social state—dyadic positivity—is associated with in-the-moment *increases* in infant RSA. Early theory characterized infant smiling as linked to abrupt decreases in arousal [37]. The current results provide a social context for this phenomenon. It was the onset of *mutual* positive expressions that was linked to increases in RSA—a physiological index of emotional arousal regulation.

The sample showed variability in social risk and prenatal drug exposure risk (see table 2). Overall, changes in RSA before the onset of bouts of positive mutual behaviours point to the sensitivity of the infant physiological system to social context generally and dyadic states specifically [38]. Notably, these results emerged while controlling for social risk and prenatal drug exposure risk. Follow-up analyses indicated that neither social risk nor prenatal drug exposure risk had direct effects on positive engagement matching [15]. However, infants with higher prenatal risk exhibited a larger RSA boost during positive social engagement matching during the reunion episode—particularly when the mother led the bout (see supplementary material, table S4 and figure S4). Positive social engagement may be a mechanism for temporary boosts in heart rate variability after stressors such as the still face. Speculatively, positive engagement matching may benefit infants who may be most in need of such a mechanism.

**Table 2.** Descriptors and risk factors (*n* = 974).

ethnicity	Hispanic	65 (6.6%)
	non-Hispanic	903 (93.4%)
race	Black	749 (77.4%)
	White	142 (14.7%)
	other	76 (7.9%)
perinatal status	gestational age (weeks)	36.21 (SD = 4.06)
	birth weight (g)	2621.32 (SD = 825.32)
prenatal drug exposure	cocaine	401 (41.5%)
	opiates	73 (7.5%)
	alcohol	568 (58.7%)
	marijuana	208 (21.5%)
	tobacco	514 (53.2%)
social risk	mother single	776 (80.4%)
	Medicaid	792 (81.9%)
	maternal education <12 years	375 (38.9%)
	<federal poverty line	573 (64.2%)

*Note*: Medicaid is a public health insurance programme that provides healthcare coverage to low-income individuals within the United States. Ethnicity and race column entries do not sum to 974 owing to missing data. Prenatal drug exposures overlap owing to polydrug exposure. Social risk exposures also overlap.

Study results provide new insight into the functions of RSA change during social interaction. The observation that RSA increases during bouts of joint positive social behaviours suggests that positive dyadic interaction allows for increases in parasympathetic system activity indexing elevated adaptability. A recent study [39] observed that across episodes of the still face paradigm, infant RSA returned to an individual-level homeostatic range, a dynamic systems set point [40]. Thus, a positive social interaction may allow the infant parasympathetic system to briefly transition to a higher level, affording a new state of behavioural flexibility for the infant and broadening momentary thought–action repertoires, expanding attention, exploration and social engagement, to help build psychological, social and cognitive resources [41–43].

Several limitations are noteworthy given the novel analytic framework used in this study. First, we used a 10-second moving window to estimate RSA time series (see §4b(v)). The 10-second moving window represents an innovation in capturing brief but stable RSA dynamics [1] as compared with window sizes of 30 seconds or greater that are common in the literature [4,13]. Moreover, given the cyclical patterning of infant respiration, a 10-second window will necessarily include multiple respiratory cycles. Nevertheless, physiological dynamics within 10 seconds of a behavioural event may be influenced by changes throughout the window. However, the more distal that physiological changes are from a behavioural onset, the more remote is the statistical influence of the time series co-occurring with the behavioural event and the greater the influence of changes occurring substantially before and after the behaviour's onset. Infant RSA, for example, rose 9 and 7 seconds before the onset of mother-led joint positive engagement bouts. Future research could profitably focus on the relations between parasympathetic dynamics and interactive behaviours using longer recording sessions—for example, multi-hour recordings. This would afford researchers the opportunity to observe longer windows of RSA dynamics around relevant behaviours.

A second limitation pertains to the rigour of the current approach. For every empirical RSA time series, we chose a random contiguous 20-second RSA time series sequence from the same infant that did not overlap the empirical time series in question. We did not control for where this 20-second time series was located in the full RSA time series. Therefore, it is possible that segments of the random time series overlapped with other empirical time series that met the criterion being tested (e.g. they may have contained mutual positive engagement). Although these bout types are low in frequency during episodes, this procedure may have biased *against* the robust results detected. Figure 1 provides a simple way to estimate which engagement states were predominant in random time series. For example, in analyses of RSA changes around behavioural bouts during the play episode, mismatch bouts occurred approximately 75% of the time. This means that randomly selected bouts from the play episode are, on average, probable to contain mismatches for three quarters of their duration. Other behavioural matches and mismatches are likely to be contained in random time series in proportion to their occurrence in a given episode. Third, although it was an unlikely occurrence, we did not control for when bouts of interest occurred within the same ±10 seconds. For example, if one bout of interest ended and another bout began within 10 seconds, part of the RSA series from both bouts would be identical. Finally, we did not include RSA time series when bouts of behaviour occurred within either the first or the last 10 seconds of an episode, which would have prevented a full analysis of their temporal dynamics. All of these potential analytic limitations, including the exploratory nature of the analyses, should be considered when interpreting the current results, providing a baseline for future related research.

We report that increases in infant parasympathetic activity were uniquely sensitive to positive social interaction. Changes in RSA did not occur in concert with infant or mother positive engagement alone. Strikingly, infant parasympathetic activity rose—diverging from random aggregate RSA series—many seconds before the onset of positive engagement-matching bouts. These results contribute to the growing understanding that the behavioural and physiological responses of infants and their partners are impacted by the context-specific nature of their emerging social interaction [38,44–46].

## 4. Methods

### (a) Lead contact

Further information and requests for resources should be directed to and will be fulfilled by the lead contact (drew.abney@uga.edu).

#### (i) Experimental model and subject details

Infant–mother dyads were participants in the Maternal Lifestyle Study [47], a multi-site longitudinal study of infants with prenatal exposure to cocaine and/or opiates and infants without cocaine/opiate exposure. Of 1388 infant–mother dyads recruited [47], 974 infant–mother dyads (4 months of age) were included in analyses for this study. Only mother–infant dyads who participated in the FFSF paradigm at 4 months (±2 weeks) and contributed behavioural and physiological data were included in analysis for this study. A social risk variable was computed by summing variables indexing maternal marital status (single), recipient of Medicaid, maternal education (below 12th grade) and below the federal poverty line. A cumulative exposure risk variable was computed by summing variables indexing fetal exposure during pregnancy to cocaine, opiate, alcohol, marijuana and/or maternal tobacco use [18]. Although this sample included risk variables that have applied relevance to the current study's hypotheses, the main rationale for using this dataset was the extremely large sample size relative to other studies in this literature area. Table 2 provides a detailed breakdown of the sample characteristics as a function of social risk and prenatal exposure.

The FFSF paradigm included three successive 2-minute episodes: play (‘play with your [baby's name] as you would at home’), still face (‘maintain a still face and look at the baby without talking, smiling, touching the baby or interacting in any way’) and reunion (‘start playing again with [baby's name]’). This particular age in human development is marked with increased reciprocal social interaction with caregivers and also increased parasympathetic responsivity. Infants’ and mothers’ behaviours were microcoded (1 Hz) using the Infant and Caregiver Engagement Phases (ICEP), which includes mutually exclusive phases of affective engagement that are coded separately for infant and mother.

### (b) Method details

#### (i) Inclusion and exclusion criteria

**Figure 6.**
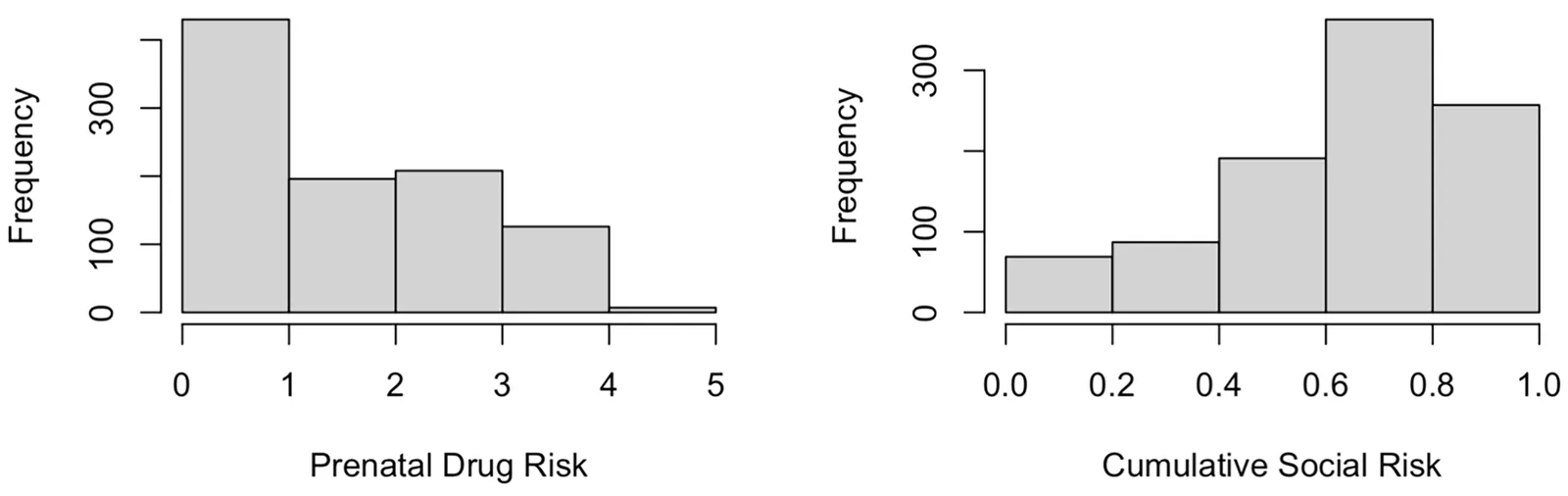
Histograms for cumulative prenatal drug risk and cumulative social risk covariates.

Our sample was comprised of infant–mother dyads of children who had various risk factors such as prenatal drug use (e.g. cocaine) and social risk (e.g. below the federal poverty line) (figure 6).

#### (ii) Experimental set-up

Dyads participated in the FFSF procedure [10]. Video rooms at each site (Brown University, University of Miami, Wayne State University and the University of Tennessee at Memphis) were identical and were equipped with an infant seat, an adjustable seat for the mother, multiple cameras, a microphone and an intercom system to communicate task instructions to the mother. Separate video streams of the upper bodies and faces of the infant and mother were merged into a multi-view display and synchronized.

#### (iii) Paradigm

Infant–mother dyads completed the FFSF paradigm, which was standardized across sites. The paradigm included a 2-minute face-to-face play episode, followed by a 2-minute episode during which the mother was instructed to keep a still face (i.e. provide no contingent responses), followed by a 2-minute reunion interaction that had the same task constraints as the first play episode.

#### (iv) Coding of data

The infant–mother dyad behaviours were coded using the ICEP [14]. The ICEP system includes a mutually exclusive set of interactive engagement phases that take into account a combination of properties like facial expressions, direction of gaze and vocalizations that form ordinal patterns (negative to positive) of social engagement. Coded behaviours for the infant engagement (passive-withdrawn, protest, object, social monitoring and social positive) and mother engagement (negative, social monitoring with no vocalizing, social monitoring with positive vocalizing and social positive) were combined to define dyadic match states (negative, neutral, positive) and mismatch states. In other words, specific individual states of infant and mother defined dyadic states. For example, if the infant was showing a ‘social positive engagement’ state simultaneously with the mother showing a ‘social positive engagement’ state, that would be coded as ‘positive engagement matching’. In another example, if the infant was showing a ‘positive-withdraw’ state and the mother was showing a ‘negative’ state, that bout would be coded as ‘negative engagement matching’. Percent agreement assessed in a random 15% of dyads was high for infant engagement (85%, *k* = 0.74) and mother engagement (84%, *k* = 0.76).

#### (v) Collection of physiological data

RSA was derived from the R–R time series (or time between adjacent R-waves) collected from a digitized electrocardiogram (ECG). ECGs were recorded using three electrodes placed on the infant's chest and abdomen. The ECG signal was sampled at 1 kHz and stored on a computer for later scoring and processing. ECG acquisition was completed on a system that recorded video images simultaneously with physiology [48], allowing for later synchronization of the audio–video and physiology recordings.

Post-processing of data was completed using a series of automated algorithms to identify R–R intervals outside of expected values. Missed or spurious R-waves were flagged and corrected via linear interpolation. A 21-point moving polynomial was then applied to remove low-frequency trends in the signal. A bandpass filter extracted the variance in heart period within the frequency band of spontaneous respiration in young children and infants (0.24–1.04 Hz) [49]. The resulting measure of RSA utilized the algorithm previously reported by Porges [35] and is in the frequency range of respiration, which is then converted into the natural logarithm and reported in units of ms squared, ln (ms)^2^. RSA time series were derived for each second of behaviour with a sliding 10-second window average. These methods have been previously reported for the MLS study [36,50,51]. RSA time series were segmented by FFSF episode and synchronized (using SMTPE timing) with the 1 Hz ICEP coding.

### (c) Quantification and statistical analysis

#### (i) Proportion of engagement matching and mismatching across infants and mothers differs as a function of FFSF episodes (analysis 1)

For each infant–mother dyad, the proportion of engagement matching (negative, neutral and positive) and engagement mismatching was computed within FFSF episodes and aggregated across the sample. Confidence intervals of ±95%were calculated to determine significant proportion differences across matching/mismatching states and across FFSF episodes.

#### (ii) Infant RSA is elevated during bouts of positive engagement matching (analysis 2)

For each bout of matched/mismatched engagement state, the average empirical RSA was computed in addition to an RSA average from a randomly chosen period of continuous RSA for a given infant during that specific episode. For each engagement matching and engagement mismatching bout, the empirical RSA average was subtracted from the random RSA average to calculate an RSA difference score where values above 0 indicated higher empirical RSA. Confidence intervals of ±95% were calculated to determine significant RSA difference score differences across matching/mismatching states and across FFSF episodes.

Mixed effects models were constructed independently for play and reunion episodes. Bout type was the main independent variable, RSA difference score was the dependent variable, and data collection clinic site, infant race, cumulative prenatal drug risk and cumulative social risk were included as covariates. Dyad ID was entered as a random intercept.

#### (iii) Infants' RSA changes as a function of the onset of bouts of engagement (analyses 3–5)

To determine differences in RSA changes as a function of the onset of a bout of engagement, we completed the following procedure:

i. RSA series were extracted ±10 seconds before and after the onset of bouts of engagement, matching for empirical series and randomly chosen 20-second segments. A new selection of random segments was conducted for each analysis (analyses 2–5).
ii. RSA series were aggregated in a long-format (21 × *b*) matrix, where *b* = the number of total bouts for that particular engagement type across the entire sample.
iii. The long-format matrix was submitted to growth curve analysis (fixed effects included: RSA type (empirical vs. random), engagement type; up to second-order orthogonal polynomials (quadratic, linear, intercept); the random effects intercept was dyad) to test differences in the RSA trajectories across empirical/random RSA types and also across engagement types. Data collection clinic site, infant race, social risk and cumulative social risk were included as covariates.
iv. Follow-up pairwise comparisons (with Bonferroni correction) testing differences between average RSA estimates across empirical and random series across engagement types were conducted at each time point (±10 seconds before and after the onset of a bout).
v. Diagnostic checks (e.g. homoscedasticity, normality of residuals) were completed to assess assumptions of regression on primary models. Q–Q plots from many of the primary models showed small deviations at the tails.

## Supplementary Material



## Data Availability

De-identified data and R code have been deposited on Open Science Framework (https://osf.io/9r3hc/overview) and are publicly available as of the date of publication. Supplementary material is available online [52].

## Theme

One contribution of 19 to a theme issue ‘Mechanisms, development, phylogeny and functions of emotional expressions’.
