# Supplementary material for: Mutual positive engagement with mother is uniquely associated with increases in human infant heart rate variability
Source: Philos Trans R Soc Lond B Biol Sci. 2026 Sep 24;381(1959):20260004. doi: 10.1098/rstb.2026.0004 (PMC13601893; doi:10.1098/rstb.2026.0004)
Supplement: Supplementary file 1 [file rstb.2026.0004.zip › rstb.2026.0004/rstb20260004_si_001.pdf]

Supplemental Analyses

Elevated Infant RSA dynamics during bouts of positive engagement matching depend on bout duration (SA 1)

Given that we observed significant increases in RSA over the course of bouts of positive engagement matching (Analysis 2) but not during bouts of neutral engagement matching or bouts of solo infant positive engagement, we investigated the impact of bout-duration on RSA dynamics. If positive social interactions impact or are impacted by physiological changes, we would expect that longer bouts of positive engagement matching would be associated with more pronounced changes in RSA dynamics, suggesting earlier and more elevated estimates of RSA before bout onset and extending after bout onset. Put another way, if matched positive engagement matching elicits increases in infant RSA, then greater magnitude RSA changes might be expected during more sustained (i.e., longer) bouts of positive engagement matching, which was the case.

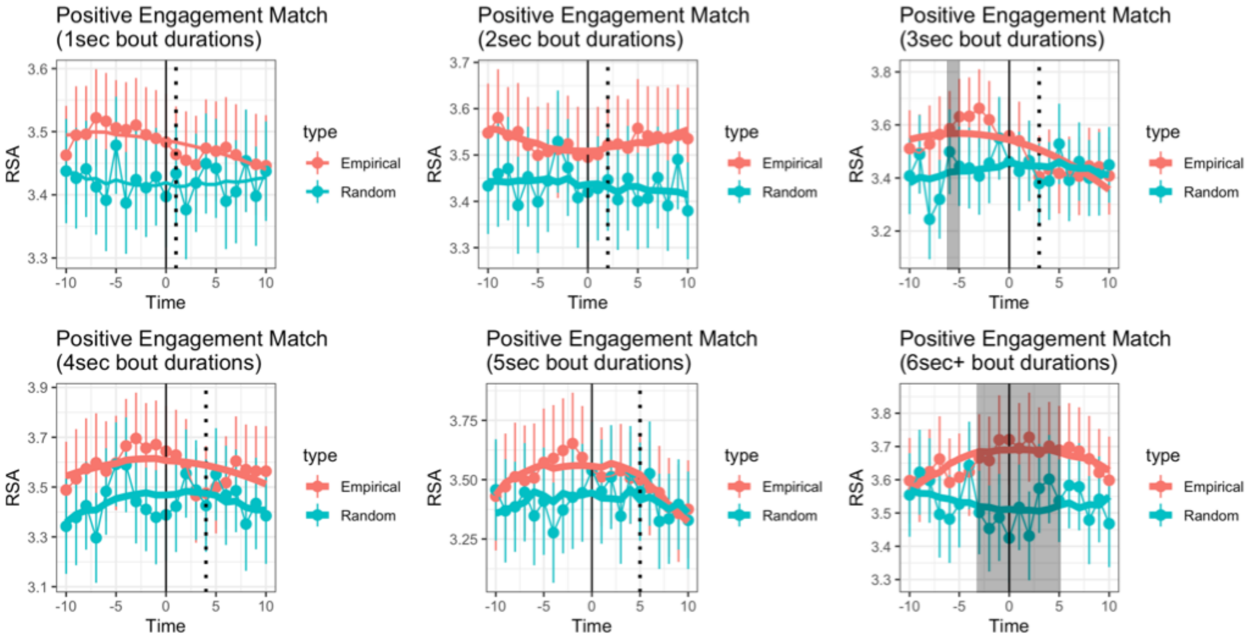

Figure S.1: RSA dynamics -/+10 before and after the onset of bouts of positive engagement matching for Empirical (red) and Random (blue) RSA series across bout duration groups during the Play episode. Solid vertical lines denote the onset of a bout. Dotted vertical lines denote bout duration. Confidence Intervals represent -/+95% intervals. Thick lines represent fitted lines from growth curve models (without covariates in model). Opaque boxes represent significant (p<.05) contrasts with Tukey corrections between RSA type at a particular time point as per the growth curve model results (including covariates in model). Note that y-axes are not identical across sub-plots.

For the Play episode, RSA series were extracted -/+10s before and after the onset of bouts of positive engagement matching for empirical series and randomly-chosen segments (Figure S.1). RSA series (Type: Empirical v. Random; Bout Duration: 1s,2s,3s,4s,5s,6s+) were submitted to

growth curve models with orthogonal polynomials to the 2nd-order (quadratic, linear, intercept). For 6s+ bouts (z-ratio=-3.37, p<.001), the 2nd-order polynomial contrast was significant suggesting a stronger negative quadratic function (low RSA to high RSA to low RSA) for the empirical series compared to the random series. Follow-up pairwise comparisons suggested differences in RSA at specific time points for only two bout durations, 3s and 6s+ bouts. For 3s bouts (note that the quadratic polynomial was not significant), there were elevated RSA estimates for the empirical series compared to the random series for two seconds (from 6s to 5s before the onset of a bout). For 6s+ bouts, there were elevated RSA estimates for the empirical series from 4s before the onset of a bout until 5s after the onset of a bout, that is, through six seconds of the positive engagement matching bout (see Figure S.1).

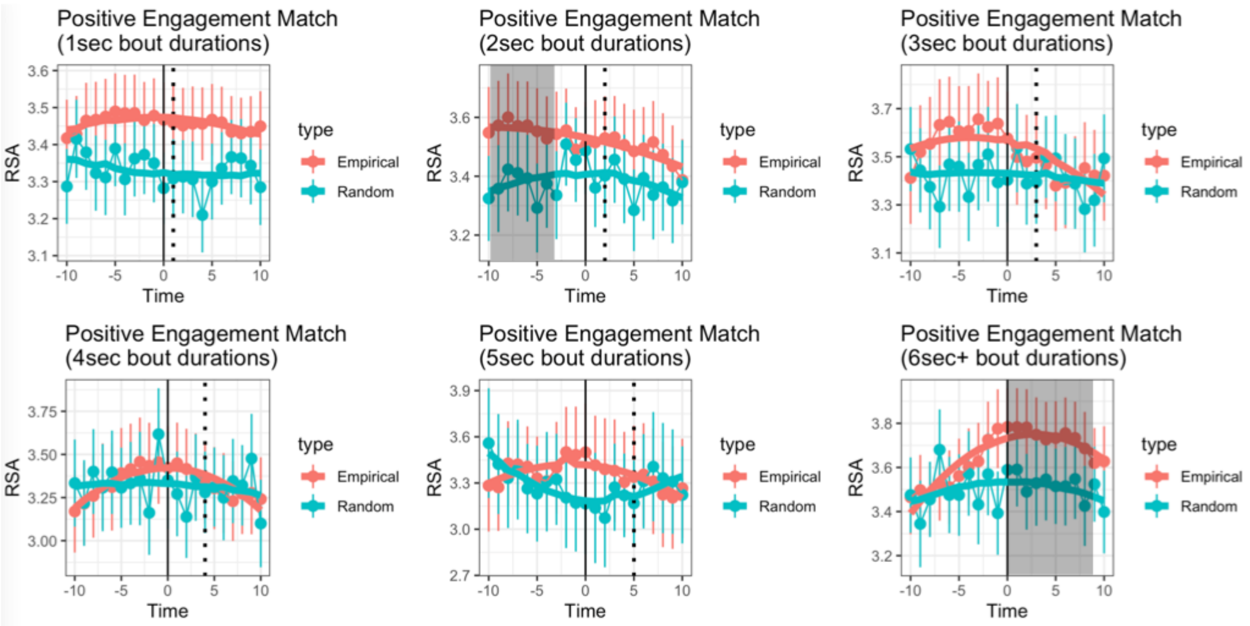

Figure S.2: RSA dynamics -/+10 before and after the onset of bouts of positive engagement matching for Empirical (red) and Random (blue) RSA series across bout duration groups during the Reunion episode. Solid vertical lines denote the onset of a bout. Dotted vertical lines denote bout duration. Confidence Intervals represent -/+95% intervals. Thick lines represent fitted lines from growth curve models (without covariates in model). Opaque boxes represent significant (p<.05) contrasts with Tukey corrections between RSA type at a particular time point as per the growth curve model results (including covariates in model). Note that y-axes are not identical across sub-plots.

For the Reunion episode, RSA series were extracted -/+10s before and after the onset of bouts of positive engagement matching for empirical series and randomly-chosen segments (Figure S.2). RSA series (Type: Empirical v. Random; Bout Duration: 1s,2s,3s,4s,5,6s+) were submitted to growth curve models with orthogonal polynomials to the 2nd-order (quadratic, linear, intercept). For 4s bouts (z-ratio=-2.53, p=.011), 5s bouts (z-ratio=-4.10, p<.001), and 6s+ bouts (z-ratio=-1.95, p=.05), the 2nd-order polynomial contrast was significant suggesting a stronger negative quadratic function (low RSA to high RSA to low RSA) for the empirical series compared to the random series. Follow-up pairwise comparisons suggested differences in RSA at specific time

points for only two different bout durations, 2s and 6s+ bouts. For 2s bouts (note that the quadratic polynomial was not significant), there were elevated RSA estimates for the empirical series from 10s before the onset of a bout until 4s before the onset of a bout. For 6s+ bouts, there were elevated RSA estimates for the empirical series at the onset of the bout until 8s after the onset of a bout (see Figure S.2).

The results from both the Play and Reunion episodes suggest that changes in RSA magnitudes depend on the duration of a positive engagement matching bout. Specifically, longer bouts of positive engagement matching were associated with (1) more RSA anticipatory dynamics and (2) higher sustained elevated levels of RSA magnitude during and after bouts. In both the Play and Reunion episodes, elevated infant RSA occurred throughout the 6 seconds that defined the minimum length of the 6s+ bouts positive engagement matching.

Elevated Infant RSA dynamics during bouts of positive engagement matching (SA 2) and positive engagement matching when mother is leading (SA 3) depend on social risk and prenatal drug exposure risk

Social risk and prenatal drug exposure risk were included as covariates in all models in the main text. A post-hoc analysis including these covariates and their interactions with positive engagement matching and linear and quadratic effects of time, quantified impacts of risk on RSA dynamics. Below are two tables that provide model output for (1) modeling the interactions between positive engagement matching, risk, and time and (2) positive engagement matching (mother leads), risk, and time.

Supplementary Table 3: Model Output for Positive Engagement Matching, Risk, and Time (SA 2)

	DF	F-Statistic	p-value
Play Episode			
Social Risk	1(475)	0.75	0.389
$Time^2$	2(217353)	12.49	<.001
Type	1(441)	37.48	<.001
Race	3(475)	2.00	0.114
Clinic	3(476)	5.17	0.002
$Time^2$*Type	2(217353)	9.57	<.001
Social Risk* $Time^2$*Type	4(217353)	2.85	0.223
Play Episode			
Prenatal Risk	1(475)	0.48	0.487
$Time^2$	2(217353)	8.55	<.001
Type	1(441)	37.48	<.001
Race	3(475)	1.89	0.130
Clinic	3(476)	5.17	0.002
$Time^2$*Type	2(217353)	11.28	<.001
Prenatal Risk* $Time^2$*Type	4(217353)	1.09	0.358
Reunion Episode			
Social Risk	1(317)	0.36	0.547
$Time^2$	2(136347)	5.97	0.003
Type	1(314)	21.95	<.001
Race	3(317)	0.90	0.441
Clinic	3(318)	3.70	0.012
$Time^2$*Type	2(136347)	1.39	0.25
Social Risk* $Time^2$*Type	4(136347)	0.95	0.435
Reunion Episode			
Prenatal Risk	1(318)	0.004	0.949
$Time^2$	2(136347)	12.26	<.001
Type	1(314)	21.95	<.001
Race	3(317)	0.83	0.479
Clinic	3(318)	3.58	0.014
$Time^2$*Type	2(136347)	6.83	0.001
Prenatal Risk* $Time^2$*Type	4(136347)	8.56	<.001

Note. Social risk is a 0-4 cumulative variable and prenatal risk is a 0-5 cumulative variable. Type refers to empirical vs. random time-series. Time refers to quadratic, linear, and intercept effects of seconds within a bout. Race and clinic are categorical variables. The Prenatal Risk* $Time^2$*Type interaction is documented in Figure S3.

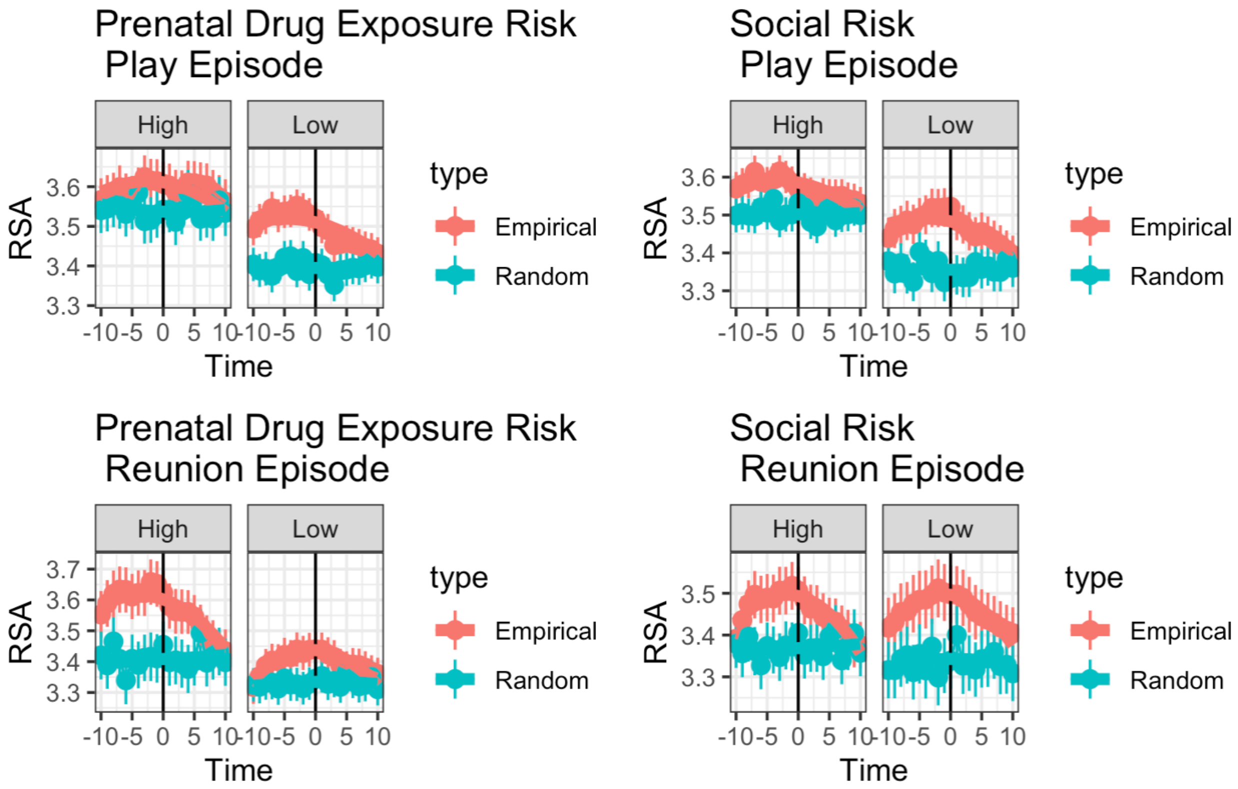

Figure S.3: RSA dynamics -/+10 before and after the onset of bouts of positive engagement matching for Empirical (red) and Random (blue) RSA series across risk levels during the Play and Reunion episodes. Solid vertical lines denote the onset of a bout. Dotted vertical lines denote bout duration. Confidence Intervals represent -/+95% intervals. Thick lines represent fitted lines from growth curve models (without covariates in model). Note that y-axes are not identical across sub-plots. Cutoff for low/high for Prenatal Drug Exposure Risk was above a score of 2 (out of 5). Cutoff for low/high for Social Risk was above a score of .50 (out of 1).

Elevated Infant RSA dynamics during bouts of positive engagement matching (mother leads) depend on social risk and prenatal drug exposure risk (SA 3)

Supplementary Table 4: Model Output for Positive Engagement Matching (mother leads), Risk, and Time

	DF	F-Statistic	p-value
Play Episode			
Social Risk	1(473)	0.90	0.344
$Time^2$	2(99972)	6.06	0.002
Type	1(421)	39.45	<.001
Race	3(474)	1.97	0.117
Clinic	3(475)	4.99	0.002
$Time^2$*Type	2(99972)	3.25	0.040
Social Risk* $Time^2$*Type	4(99972)	1.15	0.329
Play Episode			
Prenatal Risk	1(474)	0.43	0.512
$Time^2$	2(99972)	5.01	0.007
Type	1(421)	39.47	<.001
Race	3(474)	1.85	0.137
Clinic	3(475)	4.97	0.002
$Time^2$*Type	2(99972)	3.70	0.25
Prenatal Risk* $Time^2$*Type	4(99972)	0.26	0.905
Reunion Episode			
Social Risk	1(311)	0.28	0.596
$Time^2$	2(60063)	4.08	0.002
Type	1(304)	23.84	<.001
Race	3(311)	1.31	0.273
Clinic	3(312)	3.32	0.020
$Time^2$*Type	2(60063)	0.87	0.421
Social Risk* $Time^2$*Type	4(60063)	0.73	0.574
Reunion Episode			
Prenatal Risk	1(312)	0.02	0.900
$Time^2$	2(60063)	5.13	0.006
Type	1(304)	23.85	<.001
Race	3(311)	1.23	0.297
Clinic	3(312)	3.22	0.023
$Time^2$*Type	2(60063)	6.18	0.002
Prenatal Risk* $Time^2$*Type	4(60063)	7.32	<.001

Note. Social risk is a 0-4 cumulative variable and prenatal risk is a 0-5 cumulative variable. Type refers to empirical vs. random time-series. Time refers to quadratic, linear, and intercept effects of seconds within a bout. Race and clinic are categorical variables. The Prenatal Risk* $Time^2$*Type interaction is documented in Figure S4.

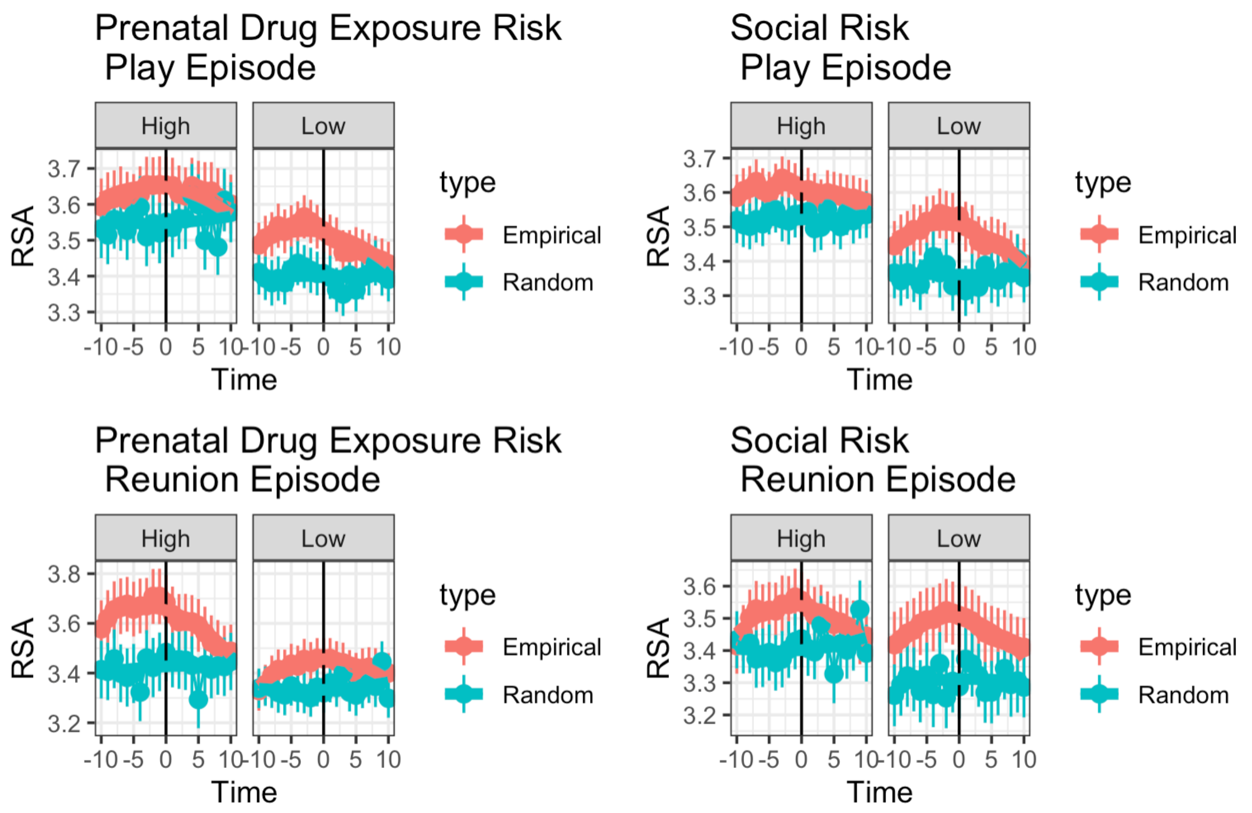

Figure S.4: RSA dynamics -/+10 before and after the onset of bouts of positive engagement matching (Mother leads) for Empirical (red) and Random (blue) RSA series across risk levels during the Play and Reunion episodes. Solid vertical lines denote the onset of a bout. Dotted vertical lines denote bout duration. Confidence Intervals represent -/+95% intervals. Thick lines represent fitted lines from growth curve models (without covariates in model). Note that y-axes are not identical across sub-plots. Cutoff for low/high for Prenatal Drug Exposure Risk was above a score of 2 (out of 5). Cutoff for low/high for Social Risk was above a score of .50 (out of 1).
